# Correction: The intramolecular hydrogen bonded–halogen bond: a new strategy for preorganization and enhanced binding

**DOI:** 10.1039/c8sc90145g

**Published:** 2018-07-24

**Authors:** Asia Marie S. Riel, Daniel A. Decato, Jiyu Sun, Casey J. Massena, Morly J. Jessop, Orion B. Berryman

**Affiliations:** a University of Montana , 32 Campus Drive , Missoula , MT , USA . Email: orion.berryman@umontana.edu

## Abstract

Correction for ‘The intramolecular hydrogen bonded–halogen bond: a new strategy for preorganization and enhanced binding’ by Asia Marie S. Riel *et al.*, *Chem. Sci.*, 2018, **9**, 5828–5836.



## 

The authors regret that in the original article there are some words missing from the sentence beginning “Studies evaluating the direct influences on…”. The sentence should read “Studies evaluating the direct influences on each other—HBing to the electron-rich belt of a XB donor or XBing to an electronegative region of a HB donor heteroatom—have been largely limited to intermolecular structural database and computational evaluations.”.

The Royal Society of Chemistry apologises for these errors and any consequent inconvenience to authors and readers.

